# Effects of pulpotomy using mineral trioxide aggregate on prostaglandin transporter and receptors in rat molars

**DOI:** 10.1038/s41598-017-07167-y

**Published:** 2017-07-31

**Authors:** Naoto Ohkura, Naoki Edanami, Ryosuke Takeuchi, Aiko Tohma, Mariko Ohkura, Nagako Yoshiba, Kunihiko Yoshiba, Hiroko Ida-Yonemochi, Hayato Ohshima, Takashi Okiji, Yuichiro Noiri

**Affiliations:** 1 Division of Cariology, Operative Dentistry and Endodontics, Department of Oral Health Science, Niigata University Graduate School of Medical and Dental Sciences, Niigata, Japan; 2 Division of Orthodontics, Department of Oral Biological Science, Niigata University Graduate School of Medical and Dental Sciences, Niigata, Japan; 3 Division of Anatomy and Cell Biology of the Hard Tissue, Department of Tissue Regeneration and Reconstruction, Niigata University Graduate School of Medical and Dental Sciences, Niigata, Japan; 4 Department of Pulp Biology and Endodontics, Graduate School of Medical and Dental Sciences, Tokyo Medical and Dental University (TMDU), Tokyo, Japan

## Abstract

Mineral trioxide aggregate (MTA) is a commonly used dental pulp-capping material with known effects in promoting reparative dentinogenesis. However, the mechanism by which MTA induces dentine repair remains unclear. The aim of the present study was to investigate the role of prostaglandin E_2_ (PGE_2_) in dentine repair by examining the localisation and mRNA expression levels of its transporter (Pgt) and two of its receptors (Ep2 and Ep4) in a rat model of pulpotomy with MTA capping. Ep2 expression was detected in odontoblasts, endothelial cells, and nerve fibres in normal and pulpotomised tissues, whereas Pgt and Ep4 were immunolocalised only in the odontoblasts. Moreover, mRNA expression of *Slco2a1* (encoding Pgt), *Ptger2* (encoding Ep2), and *Ptger4* (encoding Ep4) was significantly upregulated in pulpotomised dental pulp and trigeminal ganglia after MTA capping. Our results provide insights into the functions of PGE_2_ via Pgt and Ep receptors in the healing dentine/pulp complex and may be helpful in developing new therapeutic targets for dental disease.

## Introduction

The dentine-pulp complex possesses high repair capacity and forms reparative dentine in response to various injuries. For example, dentinal repair occurs through the activity of newly differentiated odontoblast-like cells in response to progression of caries or irritation^1^. Pulpitis is caused by further extension of an established lesion to the dental pulp, and can be clinically distinguished as either reversible or irreversible^2^. In irreversible pulpitis, the infected dental pulp tissues must be removed and replaced with root canal capping materials, such as various dental cements or gutta-percha. Teeth that have undergone endodontic treatment lose their structural integrity, sensitivity, and immune defence, which may result in extraction due to root fractures or caries. Hence, the preservation of vital pulp is important for protective resistance. Dentists demand a capping material with favourable biocompatibility and suitable physical properties in vital pulp therapy.

Mineral trioxide aggregate (MTA) is one possible choice for capping exposed pulp. MTA is mainly composed of Portland cement, which is primarily a mixture of calcium oxide and silicon dioxide^3, 4^. Many *in vitro* studies have demonstrated that MTA is biocompatible^3^, has excellent sealing ability^4^, possesses antibacterial properties^5^, induces production of pro-inflammatory mediators such as prostaglandin E_2_ (PGE_2_)^6^, and promotes angiogenesis^7^. Furthermore, MTA has been widely used for direct pulp capping to induce dentine bridge formation, which enhances pulpal protection^8^. The process of reparative dentinogenesis with MTA may principally involve the general wound healing process in the injured pulp^9^. Recent clinical application studies with histological investigation have reported that MTA is one of the best materials to repair pulp tissue with regard to the frequency of dentine bridge formation and degree of pulp inflammation^4, 10–13^. Moreover, MTA stimulates various types of cells in the pulp, including fibroblasts^14^ and osteoblast-like cells^15, 16^, to induce gene expression of mineralised tissue-related proteins such as osteopontin^14, 15^, osteonectin^14^, and osteocalcin^16^ *in vitro*. Thus, upregulation of these molecules might be involved in the reparative dentinogenic potential of MTA. However, there are insufficient data regarding the detailed mechanism of healing processes after MTA capping of exposed dental pulp.

PGE_2_ is a bioactive compound synthesised from arachidonic acid by intracellular cyclooxygenase and PGE_2_ synthase (PGES; forms include cytosolic PGES and microsomal PGES-1 and PGES-2). PGE_2_ has extensive pathophysiological effects and plays a crucial role in maintaining body homeostasis^17^. In particular, PGE_2_ is involved in inflammation, fever, and pain^17^. Transported PGE_2_ mediates pathophysiological effects, possibly via its autocrine/paracrine binding to PGE_2_ receptors (Eps) on the cell surface and signal transduction pathways^18^. At physiological pH, however, PGE_2_ predominantly exists as a charged organic anion and thus diffuses poorly through the cell membrane^19^. Carrier-mediated transport appears to compensate for its limited passive diffusion^20–22^. Prostaglandin transporter (Pgt) is one of the most studied transporters involved in the cellular release of PGE_2_^23^. We have previously reported that endothelial cells in rat incisor pulp tissue are associated with the biosynthesis of PGE_2_, and that multidrug resistance-associated protein-4 and Pgt contribute to the transport of PGE_2_ in the transmembrane pathway^20^.

The action of PGE_2_ depends largely on its binding to four types of Ep receptors (Ep1, Ep2, Ep3, and Ep4), which connect to various signal transduction pathways^24^. Binding studies have suggested that the affinity of PGE_2_ is higher for Ep4 than for Ep2^25^. Ep2 and Ep4 are both Gs-coupled receptors, which activate adenylate cyclase and induce intracellular cyclic adenylyl monophosphate (cAMP) production^26^. A recent *in vitro* study demonstrated that PGE_2_ stimulates Ep2 to mediate cAMP levels in dental pulp cells and that Ep1 and Ep3 are not involved in this process^27^. Both Ep2 and Ep4 stimulate angiogenesis and bone formation, although only Ep2 promotes cAMP-dependent neuroprotection in neurons^28^. A study of dental pulp suggested that activation of Ep receptors induces Ca^2+^ signalling to regulate cellular biological activity during inflammation^29^. However, there have been no reports on the expression and functions of Pgt, Ep2, and Ep4 during reparative dentinogenesis after MTA capping. Ep2 and Ep4 receptors, which bind to PGE_2_ transported by Pgt, may play an important role in pulpal inflammation and repair. To the best of our knowledge, however, no study has examined the localisation and functional details of the Pgt-PGE_2_-Ep pathway in dental pulp. It is paramount that clinicians be able to distinguish reversible versus irreversible pulpitis to maintain the integrity of dental pulp after injury. An understanding of the Pgt-PGE_2_-Ep pathway in the initial stage of pulpitis would provide clues to diagnose the status of pulpitis precisely. Thus, this study aimed to demonstrate the modes of expression of Pgt, Ep2, and Ep4 during reparative dentinogenesis after MTA capping, and to evaluate their functional significance (angiogenesis and neuroprotection) with gene expression assays.

## Results

### Localisation of Pgt, Ep2, and Ep4 in normal rat molar pulp

**Figure 1:**
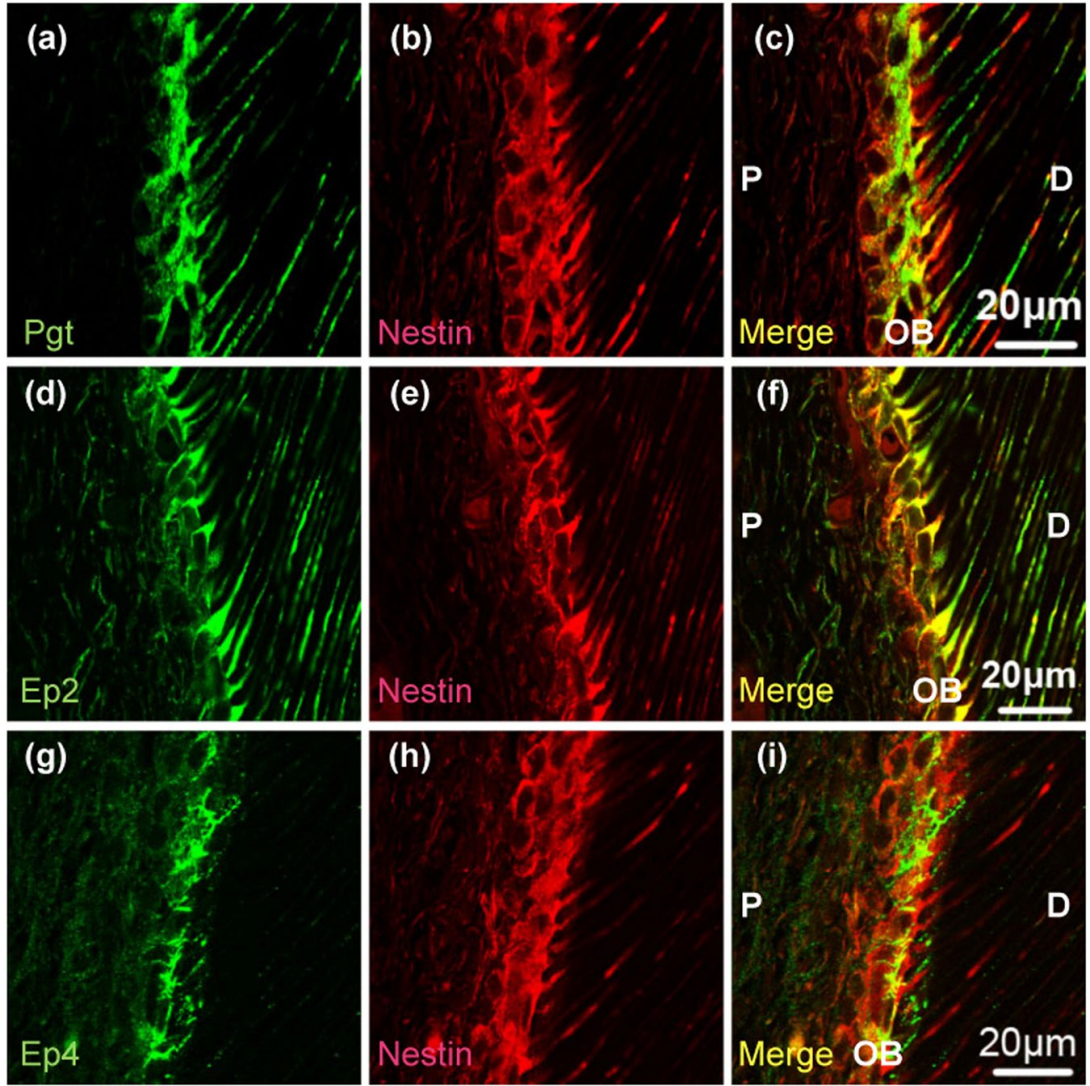
Distribution of Pgt, Ep2, Ep4, and nestin immunoreactivity in normal pulp tissue. (**a**) Pgt immunoreactivity (green). (**d**) Ep2 immunoreactivity (green). (**g**) Ep4 immunoreactivity (green). (**b**,**e**,**h**) Nestin immunoreactivity (red). (**c**,**f**,**i**) Pgt and Ep2 or Ep4 are detected in nestin-expressing odontoblasts (**c**,**f** and **i**) merged images of (**a** and **b)**, (**d** and **e**), and (**g** and **h**), respectively). Pgt and Ep2 immunoreactivities are found in the odontoblast cytoplasm and processes. In contrast, Ep4 immunoreactivity is shown exclusively in the odontoblast cytoplasm. P, pulp; OB, odontoblasts; D, dentine.

**Figure 2:**
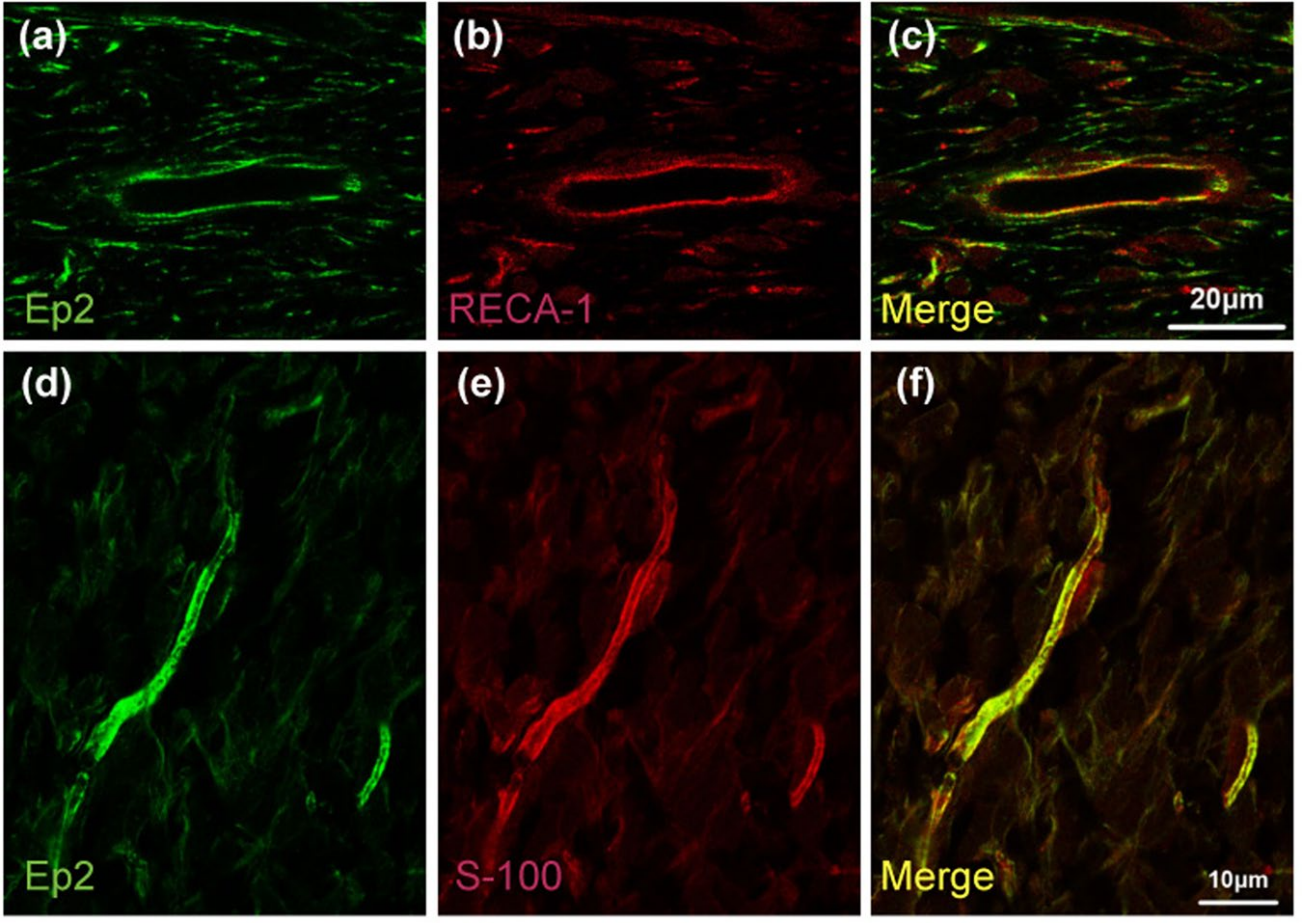
Distribution of Ep2 and RECA-1 or S-100 subunit beta immunoreactivity in normal pulp tissue. Ep2 (green) and RECA-1 (red) or S-100 subunit beta (S-100: red) immunoreactivities in normal rat molar pulp tissue. (**a**,**d**) Ep2 immunoreactivity. (**b**) RECA-1 immunoreactivity. (**e**) S-100 immunoreactivity. (**c**) Ep2 is detected in RECA-1-expressing endothelial cells (**c**: merged images of **a** and **b**). (**f**) Ep2 is detected in the cytosol of S-100-expressing Schwann cells (**f**: merged images of **d** and **e**).

The cellular expression of Pgt, Ep2, and Ep4 in the molar dental pulp was characterised with immunohistochemistry. Double immunofluorescence for nestin, a marker of odontoblasts, showed extensive overlapping with immunoreactivity for Pgt (Fig. 1a–c), Ep2 (Fig. 1d–f), and Ep4 (Fig. 1g–i). Pgt (Fig. 1a–c) and Ep2 (Fig. 1d–f) immunoreactivities were present in odontoblast cytoplasm and processes; the other pulpal cells lacked Pgt and Ep2. In contrast, Ep4 immunoreactivity (Fig. 1g–i) was found exclusively in odontoblast cytoplasm. As shown in Fig. 2, double immunofluorescence for RECA-1 (a marker of endothelial cells) and S-100 (a marker of Schwann cells) showed extensive overlapping with Ep2 immunoreactivity. Ep2 expression was found in RECA-1-positive endothelial cells (Fig. 2a–c), S-100-positive Schwann cells (Fig. 2d–f), and S-100-positive odontoblasts (data not shown).

### Localisation of Pgt and Ep2 in pulpotomised rat pulp tissue

**Figure 3:**
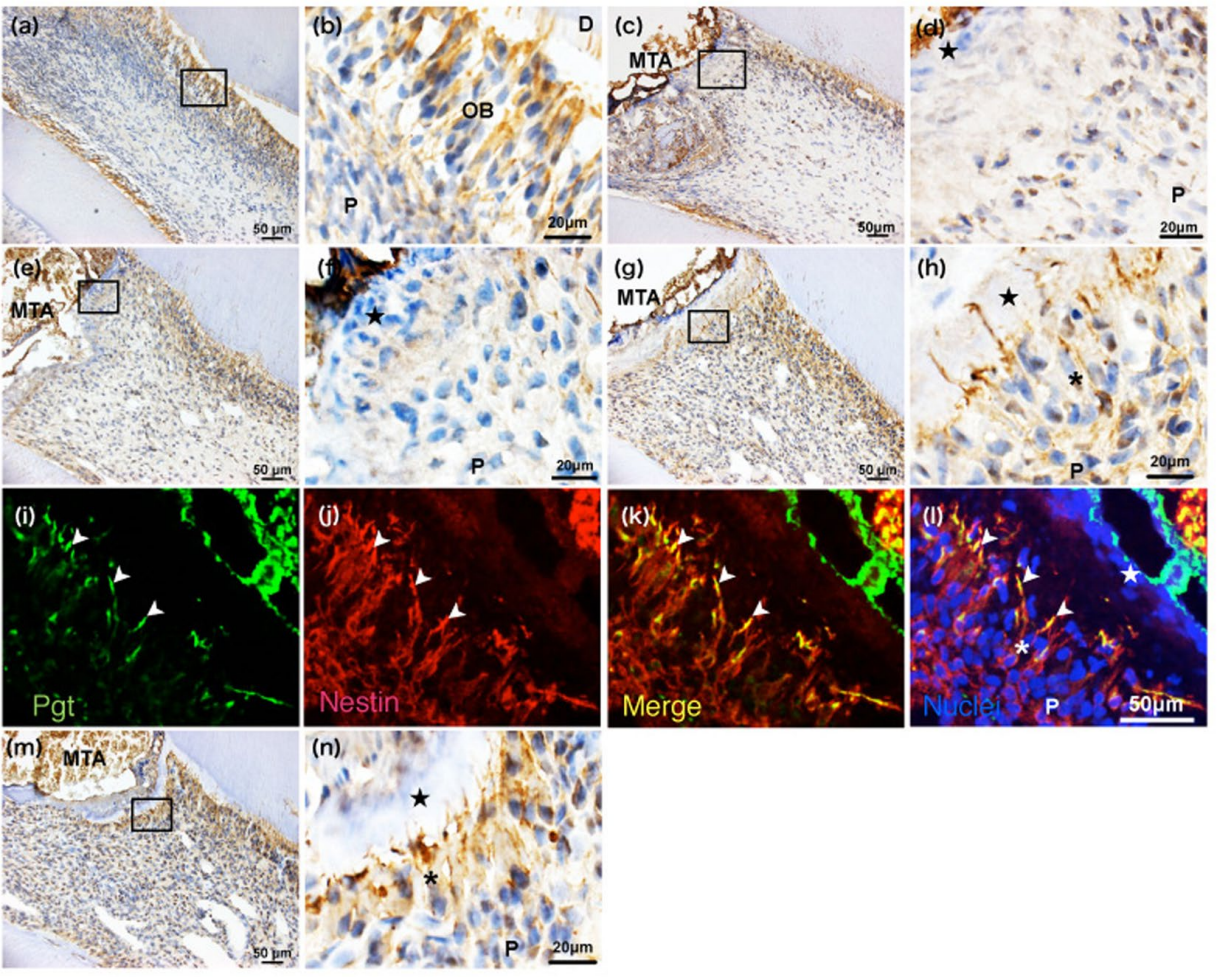
Alteration of Pgt expression over time in the pulp tissue after pulpotomy followed by MTA capping. Immunohistochemistry of Pgt in the coronal pulp tissue of normal (**a**,**b**) and injured teeth at 1 (**c**,**d**), 3 (**e**,**f**), 5 (**g**–**l**), and 7 days (**m**,**n**) after pulpotomy (n = 4 at each time point). Higher magnification of the boxed areas in (**a**,**c**,**e**,**g**, and **m**) are shown in (**b**,**d**,**f**,**h**, and **n**), respectively. Pgt immunoreactivity is not observed near the exposure site, although odontoblasts in other parts of the pulp tissue are positive for Pgt immunoreactivity at 1 day (**c**,**d**) and 3 days (**e**,**f**) after pulpotomy. (**g**–**h**) Cuboidal or columnar cells, some of which have short processes, are detected in the inner portion of the pulp at 5 days. (**i**–**l**) Pgt localisation is recognized exclusively in the odontoblast-like cell processes at 5 days after pulpotomy. (**m**,**n**) A complete dentine bridge has formed, and newly differentiated odontoblast-like cells showing Pgt immunoreactivity in their cell bodies and processes are arranged beneath the dentine bridge at 7 days. The closed star indicates the area that was exposed with a bur and capped with MTA. The asterisk indicates the odontoblast-like cell layer. The arrowhead indicates the odontoblast-like cell processes. P, pulp; OB, odontoblast; D, dentine.

At 1 and 3 days after surgery, Pgt immunoreactivity was not observed near the exposure site, although odontoblasts in other parts of the pulp tissue were positive for Pgt immunoreactivity (Fig. 3c–f). At 5 days, Pgt immunoreactivity was seen exclusively in the odontoblastic processes. Cuboidal or columnar cells, some of which had short processes, were observed in the inner portion of the pulp (Fig. 3g,h). Moreover, double immunofluorescence staining clearly detected Pgt immunoreactivity (Fig. 3i–l) on the odontoblastic processes that exhibited positivity for nestin in pulpotomised tissue after 5 days. At 7 days, a complete dentine bridge had formed, and newly differentiated odontoblast-like cells showing Pgt immunoreactivity in their cell bodies and processes were arranged beneath the dentine bridge (Fig. 3m,n).

**Figure 4:**
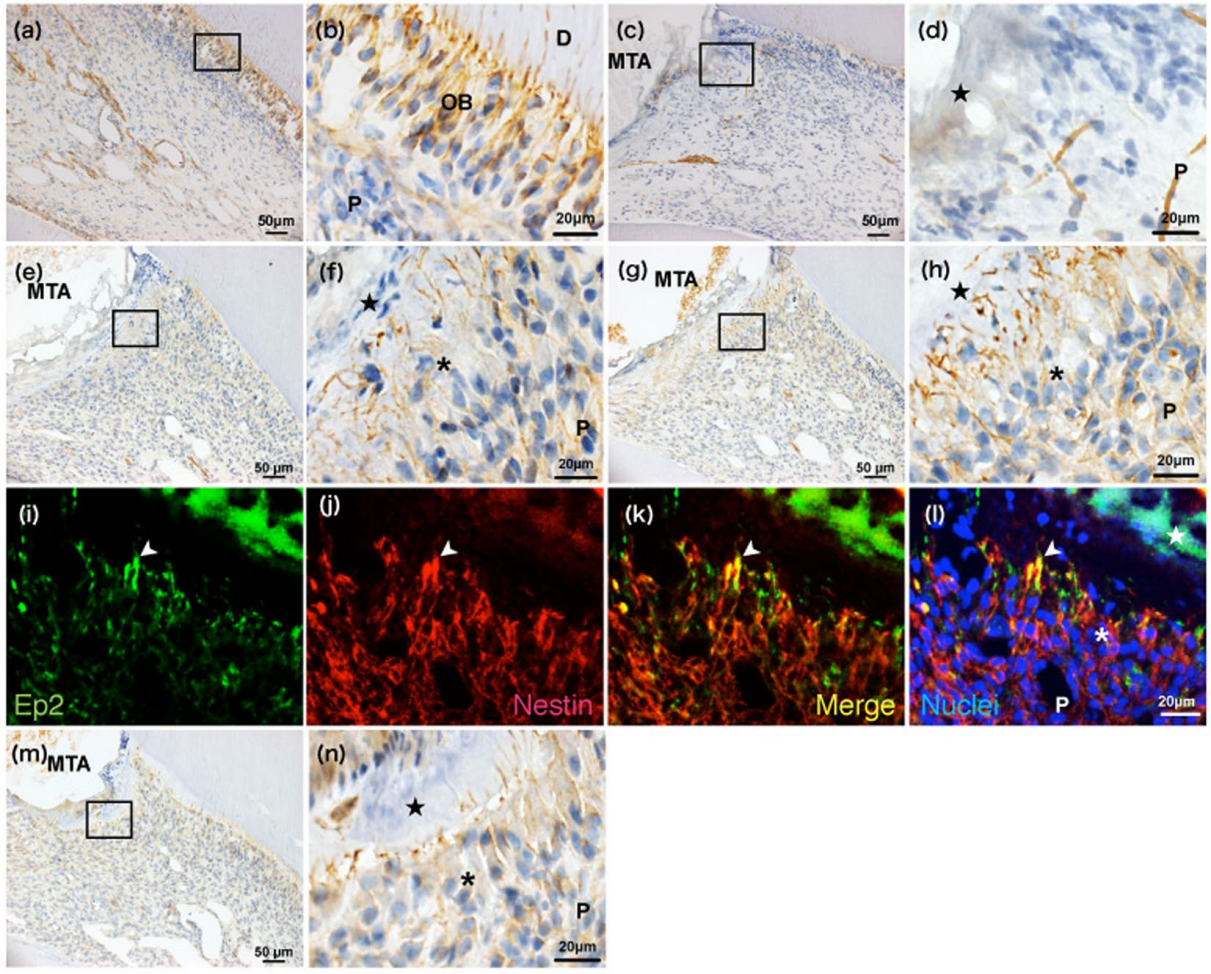
Alteration in Ep2 expression over time in the pulp tissue after pulpotomy followed by MTA capping. Immunohistochemistry of Ep2 in the coronal pulp tissue of normal (**a**,**b**) and injured teeth at 1 (**c**,**d**), 3 (**e**,**f**), 5 (**g**–**l**), and 7 days (**m**,**n**) after pulpotomy (n = 4 at each time point). Higher magnification images of the boxed areas in (**a**,**c**,**e**,**g** and **m**) are shown in (**b**,**d**,**f**,**h**, and **n**), respectively. (**c**,**d**) Ep2 immunoreactivity and new odontoblast-like cells are not detected near the exposure site, although odontoblasts in other parts of the pulp tissue are positive for Ep2 immunoreactivity at 1 day after pulpotomy. A few cells, most of which are new odontoblast-like cells, show Ep2 immunoreactivity only in the odontoblastic processes beneath the injured region at 3 days (**e**,**f**) and 5 days (**g**,**h**). (**i**–**l**) Ep2 localisation is recognized exclusively in the odontoblast-like cell processes 5 days after pulpotomy. The arrowhead indicates odontoblast-like cell processes. (**m**,**n**) At 7 days, new odontoblast-like cells showing Ep2 immunoreactivity in their cell bodies and processes are arranged beneath the reparative dentine bridge. The closed star indicates the area exposed by a bur and capped with MTA. The asterisk indicates the odontoblast-like cell layer. P, pulp; OB, odontoblast; D, dentine.

At 1 day, Ep2 immunoreactivity was not detected near the exposure site, although odontoblasts in other parts of the pulp tissue were positive for Ep2 immunoreactivity (Fig. 4c,d). At 3 and 5 days, a few cells, most of which were new odontoblast-like cells, showed Ep2 immunoreactivity only in the odontoblastic processes beneath the injured site (Fig. 4e–h). Double immunofluorescence staining clearly detected Ep2 immunoreactivity (Fig. 4i–l) on the odontoblastic processes that exhibited positivity for nestin in pulpotomised tissue after 5 days. At 7 days, new odontoblast-like cells showing Ep2 immunoreactivity in their cell bodies and processes were arranged beneath the reparative dentine bridge (Fig. 4m,n).

### mRNA expression of *Slco2a1*, *Ptger2*, and *Ptger4* in normal and pulpotomised pulp tissue

**Figure 5:**
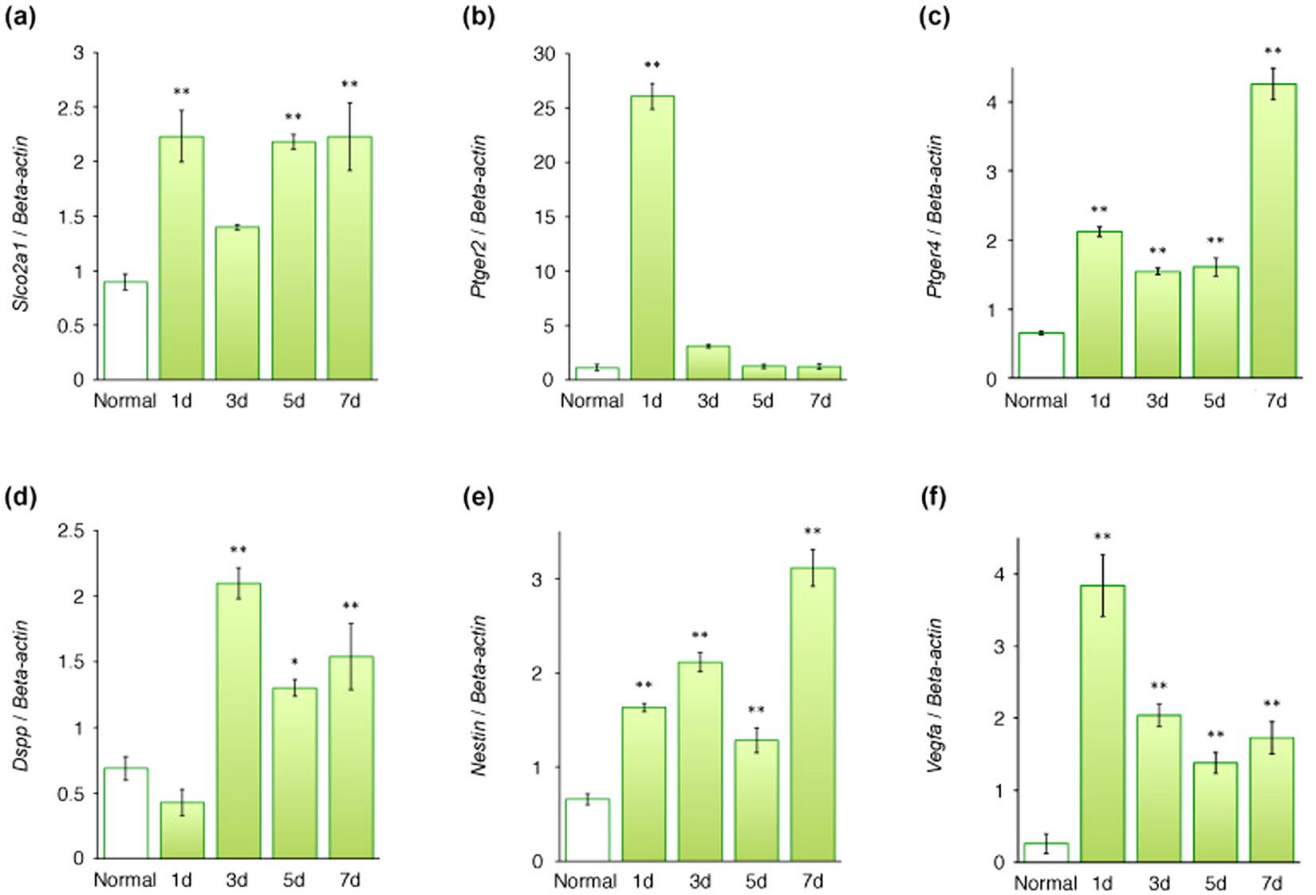
*Slco2a1*, *Ptger2*, *Ptger4*, *Dspp*, *nestin*, and *Vegfa* mRNA expression in rat first molars after pulpotomy followed by MTA capping. Real-time PCR used to quantify *Slco2a1* (**a**), *Ptger2* (**b**), *Ptger4* (**c**), *Dspp* (**d**), *nestin* (**e**), and *Vegfa* (**f**) mRNA levels in normal first molar tissue and at each examined time point after pulpotomy. Data show the mRNA expression levels of *Slco2a1*, *Ptger2*, *Ptger4*, *Dspp*, *nestin*, and *Vegfa* normalized to *β-actin* mRNA levels. (**a**) The increased expression of *Slco2a1* mRNA is maintained to 7 days. (**b**) Expression of *Ptger2* mRNA falls to normal at 3 days and thereafter. *Ptger4* (**c**), *Dspp* (**d**), and *nestin* (**e**) mRNA expression levels gradually increase in a time-dependent manner after operation. (**f**) The expression of *Vegfa* mRNA steadily decreases in a time-dependent manner, although significantly higher expression than the normal level is maintained. Bars represent mean values ± standard error of the mean, compared with the normal control (n = 6; *P < 0.05 and **P < 0.01).

As shown in Fig. 5, real-time PCR analysis showed that most mRNA examined, with the exception of *Dspp* mRNA, were significantly upregulated 1 day after surgery, compared with normal levels (P < 0.01, Fig. 5). The increased expression of *Slco2a1* mRNA was maintained to 7 days (P < 0.01, Fig. 5a). Expression of *Ptger2* mRNA fell to normal at 3 days and thereafter (Fig. 5b). *Ptger4*, *Dspp*, and *nestin* mRNA expression levels gradually increased in a time-dependent manner after pulpotomy with MTA capping (Fig. 5c–e). In contrast, the expression of *Vegfa* mRNA steadily decreased in a time-dependent manner, although significantly higher expression than the normal level was maintained (P < 0.01, Fig. 5f).

### mRNA expression of *Slco2a1*, *Ptger2*, and *Ngf* in normal and stimulated peripheral neurons

**Figure 6:**
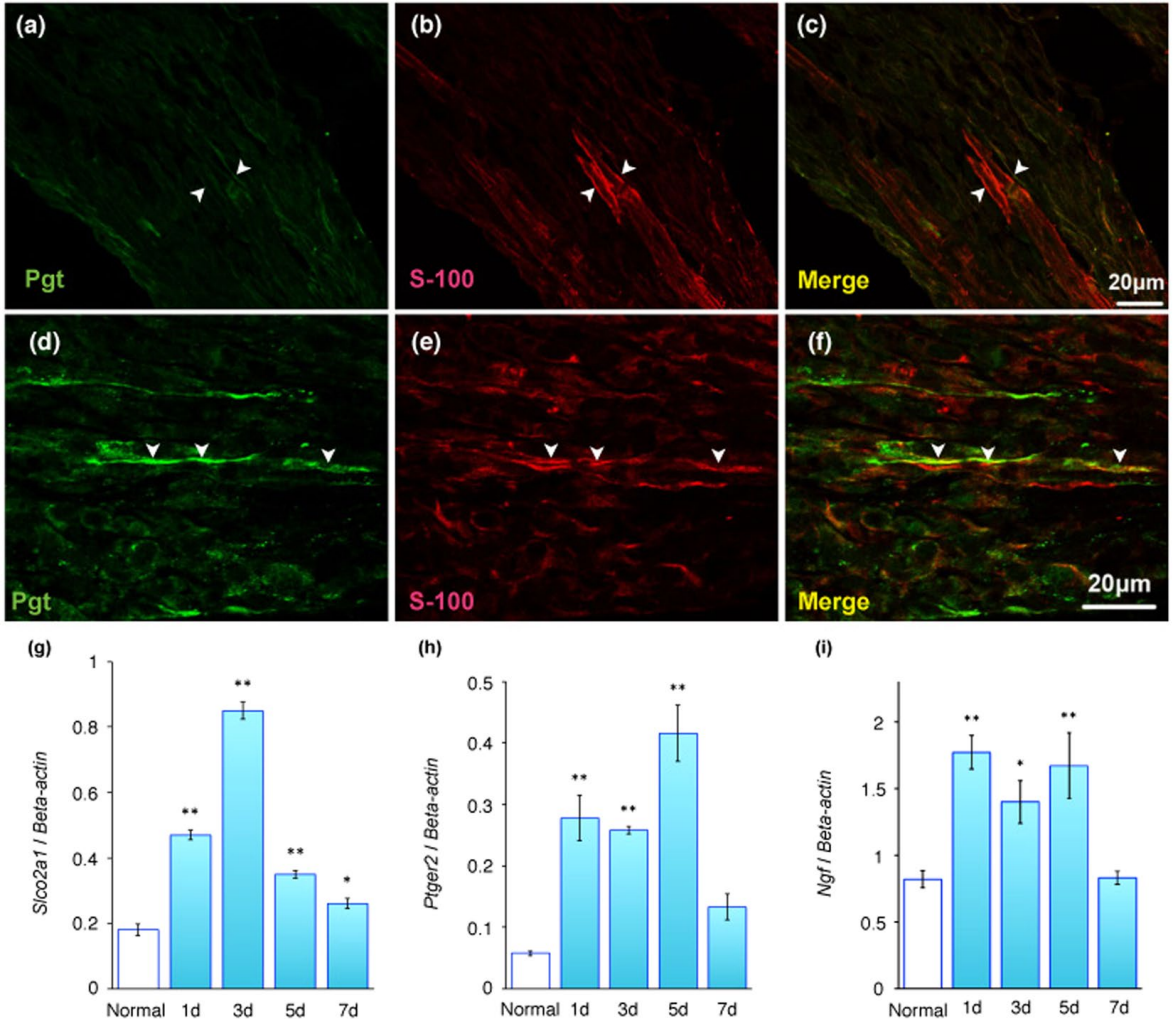
Alteration in peripheral neuron over time in pulp tissue after pulpotomy followed by MTA capping. Distribution of Pgt (green) and S-100 subunit beta (S-100; red) immunoreactivities in normal (**a**–**c**) and pulpotomised tissues 3 days after MTA capping (**d**–**f**). (**a**,**d**) Pgt immunoreactivity (green), (**b**,**e**) S-100 immunoreactivity (red). (**c**,**f**) Pgt is detected in S-100-expressing Schwann cells (**c**: merged images of **a** and **b**; **f**: merged images of **d** and **e**). Arrowheads indicate cytosol of Schwann cells. P, pulp; OB, odontoblast; D, dentine. Real-time PCR was used to quantify *Slco2a1, Ptger2*, and *Ngf* mRNA levels in normal trigeminal ganglia and at each observation point after pulpotomy. Data presented are the expression levels of *Slco2a1*, *Ptger2*, and *Ngf* mRNA normalised to *β-actin* mRNA levels. (**g**) Expression of *Slco2a1* mRNA peaks at 3 days after operation. (**h**, **i**) Expression of *Ptger2* and *Ngf* mRNA peak at 5 days after operation. Bars represent mean values ± standard error of the mean, compared with normal controls (n = 6; *P < 0.05 and **P < 0.01).

At 3 and 5 days after operation, double immunofluorescence staining clearly revealed Pgt immunoreactivity on the peripheral nerves that exhibited positivity for S-100 in the pulp tissue, whereas Pgt-positive nerves were not detected in normal pulp tissue (Fig. 6a–f). Real-time PCR analysis demonstrated increased *Slco2a1, Ptger2*, and *Ngf* mRNA levels in the trigeminal ganglion of the pulpotomised side compared with the normal trigeminal ganglion. This increase peaked at 3 to 5 days after operation (P < 0.01, Fig. 6g–i).

## Discussion

Overall, our findings indicate that the MTA-induced pulpal wound healing process is basically similar to that described in a previous report^8^; that is, the primary process of reparative dentinogenesis after MTA capping involved mild inflammatory and necrotic changes at the exposed site (Fig. 3c,d, Fig. 4c,d), followed by calcified bridge formation, which was first observed in all specimens at 7 days after treatment (Figs 3g,h and 4g,h). Because damaged pulp tissue initiated the pulpal healing process of calcified bridge formation by 7 days, we were required to observe the progress of reparative dentinogenesis beneath the MTA-capped area until 7 days to analyse the mechanism of primary inflammation and repair.

Angiogenesis is fundamental to both tissue development and wound healing^30^. Angiogenesis at sites of injury is critical for tissue repair, and proangiogenic growth factors, including VEGF, appear to be important mediators^31, 32^. VEGF is released by odontoblast-like cells^33^, dental pulp cells^34^, macrophages^35^, dendritic cells^36^, and vascular endothelial cells^37^. A previous report suggested that PGE_2_ stimulates VEGF production through EP2^38^. VEGF is known to induce angiogenesis in the dental pulp^39^. The present study showed that the time course of mRNA expression patterns for *Ptger2* and *Vegfa* were similar in pulpotomised dental pulp (Fig. 3). Moreover, the immunohistochemical analysis performed in this study demonstrated that Ep2 was localised in endothelial cells and odontoblasts in normal rat dental pulp tissue (Figs 1 and 2). Taken together, these findings indicate that Ep2 might mediate angiogenesis induced in the acute phase within the first day after dental pulp injury, in particular by affecting the function of odontoblasts and endothelial cells. Further studies of PGE_2_ signal cascades are required to elucidate the detailed mechanisms related to wound healing and pulp regeneration via Ep2.

The ability of MTA to induce reparative dentinogenesis was consistently demonstrated in a previous pulpotomy study^8^. A recent study reported that MTA pulpotomy in human permanent molars with irreversible pulpitis resulted in complete dentine bridge formation at 2 months^40^. The present study showed that *Ptger4*, *Dspp*, and *nestin* mRNA levels gradually increased in a similar time-dependent manner after MTA capping (Fig. 5c–e). However, the pattern for *Dspp* mRNA, which was not upregulated 1 day after surgery, was slightly different (Fig. 5d). Odontoblasts begin to express specific genes according to the progress of their differentiation; one such gene is *Dspp*, which encodes a noncollagenous extracellular dentine matrix protein that is cleaved into dentine sialoprotein (Dsp) and dentine phosphoprotein^41, 42^. The mRNA expression of *Dspp* was lost in the damaged odontoblasts 1 day after creation of a groove-shaped cavity in mice, and was again detectable in these cells 3 days after surgery^43^. Nestin, which is a useful odontoblast differentiation marker, was present in newly differentiated odontoblast-like cells 1 day after preparation of a groove-shaped cavity^44^. Taken together, these reports indicate that Ep4 may contribute to the differentiation of odontoblasts or odontoblast-like cells, because *Ptger4* and *nestin* mRNA expression patterns were similar. Furthermore, activation of Ep4 induced *de novo* bone formation in an experimental model of osteoporosis^45^. In the same study, Ep4-deficient mice had impaired bone formation *in vivo* in response to PGE_2_, while mice deficient in other prostaglandin receptors exhibited unchanged callus formation^45^. As explained above, these findings suggest that Ep4 might play a role in the initial stage of calcification in reparative dentinogenesis. Moreover, the present study showed that immunoreactivity for Pgt and Ep2 were localised exclusively in the cell processes of newly differentiated odontoblast-like cells during reparative dentine formation (Figs. 4 and 5). Ep2 activation stimulates local bone formation and enhances fracture-healing^46^. Moreover, both Ep2 and Ep4 mediate the anabolic functions of PGE_2_ in bone formation^47^. Overexpression of Ep2 as well as Ep4 significantly decreases lipopolysaccharide-induced chemokine (Cys–Cys motif) ligand 2 (CCL2) expression^48^. Studies have reported that CCL2 is involved in bone resorption during orthodontic tooth movement^49, 50^. Furthermore, high CCL2 expression levels were found to indicate significantly higher bone resorption activity^51, 52^. Taken together, our findings suggest that the inhibition of bone resorption through downregulation of CCL2 as a result of overexpression of Ep2 and Ep4 may induce osteogenesis (or dentinogenesis). These findings indicate that Pgt may be involved in the transmembrane efflux pathway for produced PGE_2_ during pulp repair. Moreover, some interaction of PGE_2_ via Ep2 and Ep4 might contribute to the formation of reparative dentine.

The immunohistochemical analysis performed in this study revealed that Ep2 localised in nerve fibres in normal rat dental pulp (Fig. 2d–f). Pgt localised in Schwann cells in pulp tissue 3 days after pulpotomy (Fig. 3a–f). Moreover, gene expression analysis showed that peaks in *Slco2a1*, *Ptger2*, and *Ngf* mRNA levels occurred in the trigeminal ganglion 1 to 3 days after MTA capping (Fig. 6g–h). A previous *in situ* hybridisation study reported that *Ptger2* mRNA was detected in the trigeminal ganglion^53^. Activation of Ep2 by PGE_2_ can rescue postnatal motor neurons in organotypic spinal cord slices^54^. Additionally, the Ep2 agonist butaprost can protect dopaminergic neurons from induced neurotoxicity^55^. Taken together, our findings suggest that Pgt might mediate the release of newly synthesised PGE_2_ from injured neurons, because Pgt contributes to the transport of PGE_2_ in the transmembrane pathway^20^. Moreover, these findings support the notion that PGE_2_ transported via Pgt can be neuroprotective in repair after pulpotomy.

The present study provides novel insights into the putative intracellular pathway of PGE_2_ in dental pulp cells during pulpal healing following pulpotomy. Odontoblasts with Pgt and Ep2 immunoreactivity throughout their cytoplasm, including in the cellular processes, lost their immunoreactivity after pulpotomy; newly differentiated odontoblast-like cells acquired Pgt and Ep2 immunoreactivity in their cellular processes. Previous *in vitro* studies demonstrated that MTA significantly increased PGE_2_ production in dental pulp cells in a time-dependent manner^6^ and that Pgt was involved in the efflux of intracellularly produced PGE_2_ into the extracellular milieu^23^. Taken together, these findings indicate that PGE_2_ released by Pgt stimulates odontoblast differentiation during MTA-induced pulpal wound healing in an autocrine/paracrine manner by binding to Ep2/Ep4.

In conclusion, Ep2 expression was detected in odontoblasts, endothelial cells, and nerve fibres; both Pgt and Ep4 were immunolocalised to the odontoblasts. Moreover, mRNA expression of *Slco2a1*, *Ptger2*, and *Ptger4* was significantly upregulated in pulpotomised dental pulp and trigeminal ganglia after MTA capping. The present findings provide novel insights into the functions of PGE_2_ via Pgt and Ep2/Ep4 receptors in the pulp tissue and may be helpful in the development of new therapeutic target for the treatment of deep caries.

## Materials and Methods

All animal experiments were conducted in compliance with a protocol that was reviewed by the Institutional Animal Care and Use Committee of Niigata University and approved by the President of Niigata University (Permit Number: #27 Niigata Univ. Res.79-3).

### Pulpotomy procedure

Forty 8-week-old male specific-pathogen-free Wistar rats (Charles River, Yokohama, Japan) were used in this study. Rats were housed in plastic cages in a colony room with a 12 hour light/dark cycle and an ambient temperature maintained at 25 °C. Standard pellet chow and water were available ad libitum. Under anaesthesia with an intraperitoneal injection of pentobarbital sodium (30 mg/kg), the pulp of the upper left first molar was exposed and pulpotomised through the occlusal surface with a #1 round carbide bur (ISO number 1/008; diameter, 0.8 mm). The exposed area was rinsed with 5% sodium hypochlorite (Neocleaner; Neo Dental Chemical Products, Tokyo, Japan) followed by sterile saline. Haemorrhage was controlled with sterile cotton pellets. The exposed pulp was then capped with MTA (white ProRoot MTA; Dentsply Tulsa Dental, Tulsa, OK), mixed according to the manufacturer’s instructions. MTA was placed over the pulp stump, and the cavities were sealed with a flowable composite resin (Beautifil Flow; Shofu, Kyoto, Japan). These tissues were prepared in the same way for later immunohistochemical staining and gene expression analysis. The upper right first molar and trigeminal ganglion of the same animal were used as controls. Observation points were set at 1, 3, 5, and 7 days after operation (n = 10 each).

### Immunohistochemical staining

The rats were lightly anaesthetised with 2% isoflurane in oxygen and then deeply anaesthetised with an intraperitoneal injection of 10% chloral hydrate (1 mL/250 g body weight) at selected time points. Then the animals received a transcardial perfusion of phosphate-buffered saline (PBS) containing heparin followed by 4% paraformaldehyde as fixative for 10 minutes. The relevant teeth were removed together with the surrounding tissue and immersed in the same fixative for an additional 24 hours. After demineralisation in a 10% ethylenediaminetetraacetic acid solution for 4 weeks, the specimens were cryoprotected in 10% sucrose followed by 20% sucrose in 0.01 mol/L PBS, embedded in an embedding medium (O. C. T. Compound; Sakura Finetek, Torrance, CA), frozen in liquid nitrogen, and kept at −30 °C until evaluation. Serial sagittal sections were cut at a thickness of 8 μm and processed for immunohistochemistry.

The sections were heat pretreated in 10 mmol/L citric acid buffer (pH = 6.0) at 70 °C for 20 minutes for antigen retrieval and then treated with 0.3% hydrogenous peroxidase in methanol for 30 min at room temperature to block endogenous peroxidase activity. After being rinsed in PBS, the samples were processed for immunohistochemistry using the antibodies with 5% skim milk in PBS for 1 hour at room temperature to block non-specific protein-binding sites. For 3,3′diaminobenzidine (DAB) staining, the expression of Pgt and Ep2 was examined after overnight exposure to primary antibody (rabbit anti-Pgt, 1:100; Alpha Diagnostic International, San Antonio, TX and rabbit anti-Ep2; 1:250, Abcam, Cambridge, UK), and then to the corresponding secondary biotinylated goat anti-rabbit antibody for 1 hour at room temperature (goat anti-rabbit immunoglobulin G [IgG], 1:200: Dako, Glostrup, Denmark). Finally, the samples were stained with a DAB substrate kit (Dako), counterstained with haematoxylin, and examined microscopically. Immunohistochemical negative control was made by replacing the primary antibodies with PBS; this showed no specific immunoreaction (Supplementary Fig. 1a). Digital images were taken with a CCD camera attached to a microscope (Eclipse E800; Nikon).

For immunofluorescent double-labelled staining, the sections were washed in PBS and then incubated for 24 hours at 4 °C with a cocktail of mouse anti-rat nestin (an odontoblast marker, diluted 1:100; Millipore Corporation, Darmastad, Germany) and one of the following polyclonal antibodies: rabbit anti-Pgt (1:100, Alpha Diagnostic International), rabbit anti-Ep2 (1:250, Abcam), or rabbit anti-Ep4 (1:100, Abcam). Mouse anti-rat endothelial cell antigen-1 (RECA-1; 1:50, BioRad, Hercules, CA) and anti-S100 (1:2000, Sigma-Aldrich, St. Louis, MO) antibodies were used to identify endothelial cells and Schwann cells, respectively. After being rinsed with PBS, the sections were further incubated for 1 hour with a mixture of goat anti-rabbit IgG antibody-conjugated AlexaFluor 488 (1:200, Thermo Fisher Scientific, Waltham, MA) and goat anti-mouse IgG antibody-conjugated AlexaFluor 546 (1:200, Thermo Fisher Scientific) with 4’6-diamidino-2-phenylindoledihydrochloride (DAPI; Vector Laboratories, Burlingame, CA) staining for the nucleus. Immunohistochemical negative controls were performed by replacing the primary antibody with PBS; these showed no specific immunoreaction (Supplementary Fig. 1b–e). The control sections did not exhibit any specific immunoreactivity. Digital images were taken with a CCD camera attached to a confocal laser scanning microscope (IX71; Olympus, Tokyo, Japan) or an epifluorescence microscope (Eclipse E800; Nikon). To create dual-colour or triple-colour images, the images of the same field obtained with fluorochrome were merged using image-processing software (Photoshop CS3; Adobe, San Diego, CA).

### Gene expression assay

Total RNA was isolated from the first molar or trigeminal ganglion using a TRIzol^®^ reagent (Thermo Fisher Scientific), according to the manufacturer’s instructions. To study mRNA expression in peripheral nerves in the dental pulp, we adopted the method of extracting the trigeminal ganglion that houses the central cell body^56^. Single strand cDNA was prepared from 0.5 μg of total RNA with reverse transcriptase by using a PrimeScript RT Master Mix (Perfect Real Time; Takara Bio Inc., Otsu, Japan). Real-time PCR was performed with a GeneAmp PCR system 7900 HT (Applied Biosystems, Foster City, CA) with SYBR Premix Ex Taq II (Perfect Real Time; Takara Bio Inc.), according to the manufacturer’s protocol. To quantify the amount of specific mRNA in the samples, a standard curve was produced for untreated first molars and trigeminal ganglia. This enabled standardisation of the initial mRNA content of cells relative to the amount of *β-actin*. PCR was performed by using specific primers for rat *Slco2a1*, *Ptger2* (the Ep2 receptor gene), *Ptger4* (the Ep4 receptor gene), *vascular endothelial growth factor* (*Vegfa*), *dentine sialophosphoprotein* (*Dspp*), *nestin*, *nerve growth factor* (*Ngf*), and *β-actin*. The sequences of all primers are shown in Supplementary Table 1.

Vegfa is an angiogenesis marker^31, 32^. Ngf is a neurotrophin family protein that promotes neurite outgrowth^57^ and neuronal differentiation^58^. In addition, Ngf alters trigeminal ganglion sensory neuron survival *in vitro* ^59^. Ngf is upregulated in inflamed dental pulp^60^ and plays a crucial role in the symptom of hyperalgesia following inflammation and nerve injury^61, 62^. Thus, understanding the mRNA expression patterns of *Ngf* during neuroprotection would provide informative data that could contribute to further investigation of neuroprotection.

### Statistical analysis

Data were analysed with one-way analysis of variance and Dunnet’s test using statistical software (SPSS 10J for Windows; SPSS Japan, Tokyo, Japan) and compared with untreated tissue at a significance level of 1 or 5%.

## Electronic supplementary material

Supplementary dataset
