# Supplementary material for: Effects of pulpotomy using mineral trioxide aggregate on prostaglandin transporter and receptors in rat molars
Source: Sci Rep. 2017 Jul 31;7:6870. doi: 10.1038/s41598-017-07167-y (PMC5537257; doi:10.1038/s41598-017-07167-y)
Supplement: Supplementary file 1 — Supplementary dataset [file 41598_2017_7167_MOESM1_ESM.pdf]

Supplementary Figure and Table

Effects of pulpotomy using mineral trioxide aggregate on prostaglandin transporter and receptors in rat molars.

Naoto Ohkura[1*], Naoki Edanami[1], Ryousuke Takeuchi[1], Aiko Tohma[1], Mariko Ohkura[2], Nagako Yoshiba[1], Kunihiko Yoshiba[1], Hiroko Ida-Yonemochi[3], Hayato Ohshima[3], Takashi Okiji[4], Yuichiro Noiri[1]

Affiliations

[1]Division of Cariology, Operative Dentistry and Endodontics, Department of Oral Health Science, Niigata University Graduate School of Medical and Dental Sciences, Niigata, Japan

[2]Division of Orthodontics, Department of Oral Biological Science, Niigata University Graduate School of Medical and Dental Sciences, Niigata, Japan

[3]Division of Anatomy and Cell Biology of the Hard Tissue, Department of Tissue Regeneration and Reconstruction, Niigata University Graduate School of Medical and Dental Sciences, Niigata, Japan

[4]Department of Pulp Biology and Endodontics, Graduate School of Medical and Dental Sciences, Tokyo Medical and Dental University (TMDU), Tokyo, Japan

*Correspondence: ohkura@dent.niigata-u.ac.jp

Supplementary Figure 1.

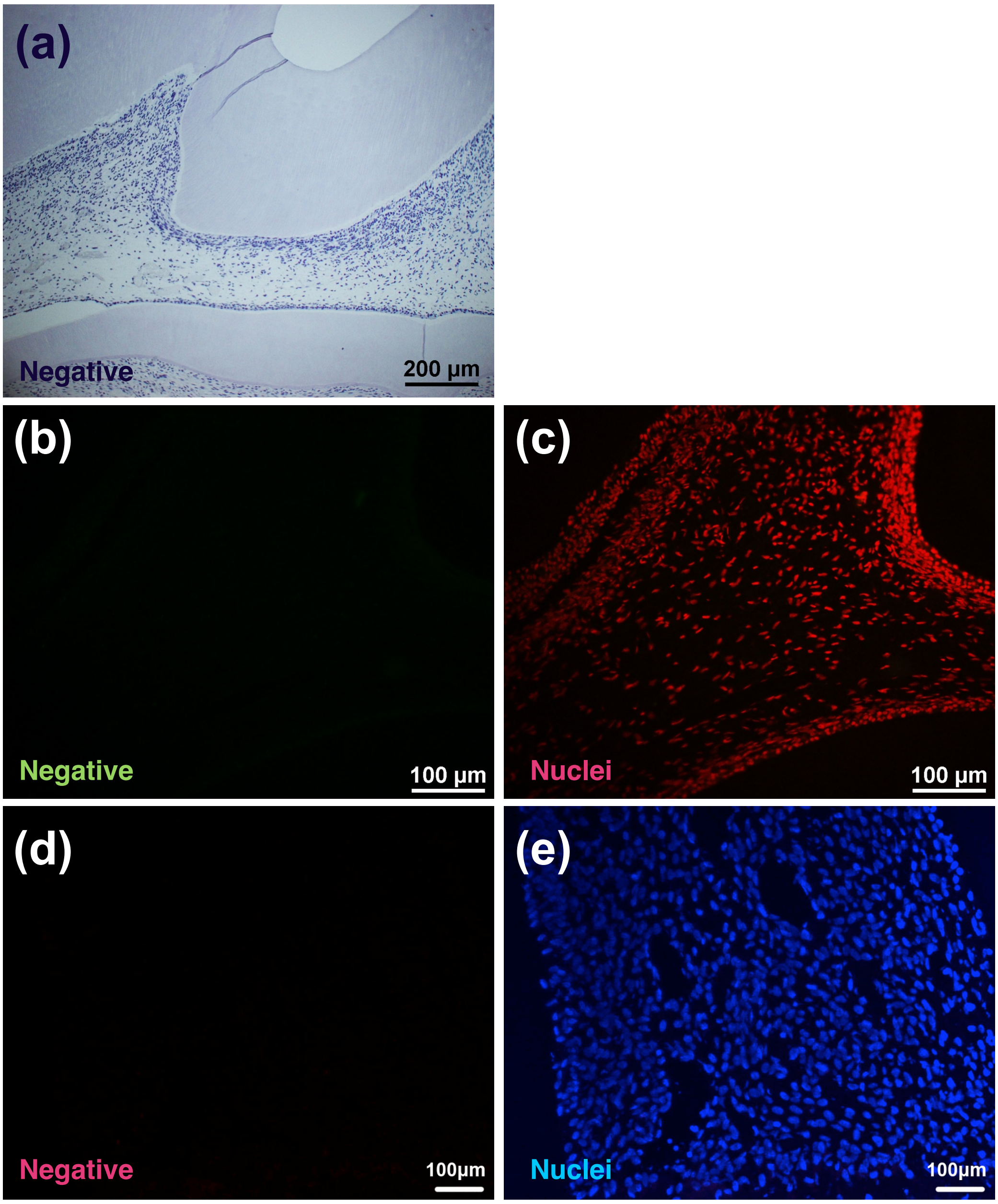

Negative control staining for antibody validation

Negative control staining was performed by replacing both primary rabbit antibodies with PBS. (**a**) Negative control for 3,3’ diaminobenzidine staining shows no specific immunoreaction. (**b**, **d**) The control sections do not exhibit any specific immunoreactivity. (**b**) Goat anti-rabbit IgG antibody-conjugated AlexaFluor 488 (green) for secondary antibody. (**d**) Goat anti-mouse IgG antibody-conjugated AlexaFluor 546 (red) for secondary antibody. (**c**) Propidium iodide (PI: red) is detected in nuclei. (**e**) 4’6-diamidino-2-phenylinodole (DAPI: blue) is detected in nuclei.

Supplementary Table 1. Primers used in this study

Moleculer name	Accessin NO.	Primer		
		Name of oligomer	Sequence	Expected size
Slco2a1	NM_022667	Sense primer	gccagatacccacaaggaga	166bp
		Anti-sense primer	gatggcgaataggatggaga	
Ptger2	NM_031088	Sense primer	cagcttcggagcaaaagaag	129bp
		Anti-sense primer	cataatggccaggagaatgagg	
Ptger4	NM_032076	Sense primer	gtcatcttactcatcgccacct	139bp
		Anti-sense primer	ttctgatggcctgcaaatcc	
Vegfa	NM_001110333.2	Sense primer	aacctcaccaaagccagcac	125bp
		Anti-sense primer	ttgaccctttccctttcctc	
Dspp	NM_012790	Sense primer	cggtattgaagaaggcgatg	122bp
		Anti-sense primer	atccctgattttggctctgc	
Nestin	AF538924.1	Sense primer	tccttagccacaaccctcaac	109bp
		Anti-sense primer	agatttgcccctcatcttcc	
Ngf	NM_001277055.1	Sense primer	aaccaatagctgcccgtgtg	139bp
		Anti-sense primer	aaatccagagtgtccgaagagg	
β-actin	NM_031144.2	Sense primer	cagggtgtgatggtgggtat	146bp
		Anti-sense primer	gtgtggtgccaaatcttctc	
